# Hepatitis B Virus Genotype Distribution and Genotype-Specific BCP/preCore Substitutions in Acute and Chronic Infections in Argentina

**DOI:** 10.1371/journal.pone.0121436

**Published:** 2015-03-30

**Authors:** María Mora González López Ledesma, Laura Noelia Mojsiejczuk, Belén Rodrigo, Ina Sevic, Lilia Mammana, Omar Galdame, Adrian Gadano, Hugo Fainboim, Rodolfo Campos, Diego Flichman

**Affiliations:** 1 Cátedra de Virología, Facultad de Farmacia y Bioquímica, Universidad de Buenos Aires, Buenos Aires, Argentina; 2 Unidad de Hepatología, Hospital de Infecciosas “F. Muñiz,” Buenos Aires, Argentina; 3 Unidad de Hepatología, Hospital Italiano Buenos Aires, Buenos Aires, Argentina; University of Cincinnati College of Medicine, UNITED STATES

## Abstract

**Aim:**

In order to assess Hepatitis B Virus genotype (g) and subgenotype (sg) implications in the course of infection, 234 HBsAg positive patients in different infection stages were characterized (66 acute infections, 63 HBeAg positive chronic infections and 105 anti-HBe positive chronic infections).

**Results:**

Overall, sgA2 (17.9%), gD (20.9%), sgF1b (34.2%) and sgF4 (19.7%) were the most prevalent. Subgenotype F1b was overrepresented in acute and chronic HBeAg infections (56.1%), whereas gD was the most frequent (40.0%) in anti-HBe positive chronic infections. Among chronic infections, HBeAg positivity rates were 50.0, 12.5, 62.8 and 35.3% for sgA2, gD, sgF1b and sgF4, respectively (p <0.05). A bias toward BCP/preCore mutations was observed among genotypes. In anti-HBe positive chronic infections, sgF1b was more prone to have A1762T/G1764A mutation than sgA2, sgF4 and gD (75.0, 40.0, 33.3 and 31.8%, p<0.005), whereas in the pC region, gD and sgF4 were more likely to have G1896A than sgA2 and sgF1b (81.0, 72.7, 0.0 and 31.3%, p <0.001). The unexpected low frequency of the G1896A mutation in the sgF1b (despite carrying 1858T) prompted us to perform a further analysis in order to identify genotype-specific features that could justify the pattern mutations observed. A region encompassing nucleotides 1720 to 1920 showed the higher dissimilarity between sgF1b and sgF4. Genotypes and subgenotypes carrying the 1727G, 1740C and 1773T polymorphisms were prevented to mutate position 1896.

**Discussion:**

HBeAg seroconversion is a critical event in the natural history of HBV infection. Differences in the HBeAg positivity rate might be relevant since different studies have observed that delayed HBeAg seroconversion is associated with a more severe clinical course of infection, highlighting the critical role that genotypes/subgenotypes might play in the progression of HBV infection. Polymorphisms in the regions 1720 to 1920 could be involved in the molecular mechanisms underlying seroconversion of each genotype/subgenotype.

## Introduction

Worldwide, hepatitis B virus (HBV) infection is a major health problem. More than 2 billion people have been infected with HBV and about 350 million individuals remain chronically infected [1–4]; they constitute an enormous virus pool, a source of infection for susceptible hosts, and most importantly, a population with high morbidity and mortality due to chronic liver disease, including hepatocellular carcinoma (HCC) [5,6]. In addition, it is estimated that four million new acute HBV infections occur every year [7].

HBV causes self-limited and persistent infections; the outcome of primary infections depends on the balance between host immunity and viral survival strategies [8]. The signs and symptoms of acute hepatitis B usually subside within 6 months, accompanied by viral clearance; the infection is defined as chronic when it persists for more than 6 months [9,10].

Acute HBV infection may lead to spontaneous clearance of the virus within weeks or months, or less frequently to acute liver failure or fulminant hepatitis. By contrast, chronic HBV infection implies persistent viral replication and is associated with risk of progression to liver cirrhosis (LC) or HCC [2,11].

The evolution of HBV infection depends on the interaction between the virus and the host’s immune response [12]. Among viral factors, i-HBV genotypes and ii- viral heterogeneity in the Basal Core Promoter (BCP) and preCore (pC) regions, which are associated with the regulation and expression of hepatitis B e antigen (HBeAg), have been widely characterized.

The preCore region codes for the preCore protein, a precursor for the secreted HBeAg. In chronic carriers, a spontaneous seroconversion can occur, with the development of antibodies to this antigen (anti-HBe) and the subsequent loss of circulating HBeAg.

Although HBeAg is not required for viral replication, it appears to be necessary for establishing viral persistence in animal models [13]. It is considered to be a tolerogen that buffers the immune attack on the infected hepatocytes [14,15]. The presence of mutations affecting HBeAg expression during the acute stage is associated with more severe clinical courses and/or fulminant hepatic failure [16–20]; furthermore, in contrast to acute HBV infections with wild-type HBV, infections with pC mutant rarely, if ever, go into chronicity [21].

HBV has been classified into eight main genotypes (designated A-H) [22–24], and two additional genotypes (I and J) were tentatively proposed [25–27]. HBV genotypes have been further separated into several subgenotypes that differ by 4.0 to 7.5% in the whole nucleotide sequence [28].

HBV genotypes differ substantially in many virological and probably some clinical parameters; however, the precise role of HBV genotypes in the evolution of the infection remains controversial. Due to geographical distribution, only two or three HBV genotypes co-circulate in most regions of the world, thereby limiting genotype comparisons [14,29].

Moreover, HBV genotypes influence the mutation pattern in the BCP and pC regions and therefore the HBeAg seroconversion rate [30,31]. Based on the stability of the encapsidation signal (nt 1847–1907) it has been established that genotypes carrying 1858T (B, C2, C3, D, E, F1 and F4) favor the emergence and selection of G1896A, whereas the double mutation A1762T/G1764A in the BCP is more prone to occur in those genotypes carrying 1858C (A, C1, F2, F3 and H) [14,32].

Notwithstanding this, the role of mutations in the BCP and pC regions in the evolution of acute and chronic infections is still controversial.

Therefore, the aim of this study was to assess the prevalence of mutations and their relationship with the viral genotype in patients with acute and chronic HBV infections.

## Patients and Methods

### Patients

This cross-sectional study included 234 untreated HBsAg positive patients, admitted to the Hepatology Unit of the Hospital Italiano de Buenos Aires and to the Hospital de Infecciosas "F. Muñiz" de Buenos Aires, and recruited during 2004–2013.

Diagnostic criteria for acute infection (AHB) was as follows: acute onset of symptoms without history of chronic HBV infection, levels of serum alanine aminotransferase (ALT) >10-fold the upper reference limit, positivity for IgM antibody to the hepatitis B core antigen (anti-HBc), rapid drop of HBsAg titer, serum HBV-DNA elimination and HBeAg seroconversion at convalescent phase. The diagnosis was confirmed by HBsAg clearance within 6 months after the initial onset; alternatively, when serum HBsAg had persisted for at least 6 months, after the onset of clinically acute hepatitis, diagnosis of acute infection was assessed by liver biopsy.

Chronic infections (CHB) met the following criteria: HBsAg positivity for more than 6 months, a history of chronic hepatitis based on a histo-pathological diagnosis and/or compatible laboratory data and ultrasonographic findings.

Patients were excluded if they had any evidence of autoimmune hepatitis or markers of hepatitis C virus, hepatitis D virus or human immunodeficiency virus.

Patients were divided into three groups: AHB, 66 patients with acute HBV infection; CHB HBeAg positive, 63 chronic patients who were HBeAg positive at baseline; CHB anti-HBe positive, 105 chronic patients who were persistently HBeAg negative.

### HBV-DNA amplification (S and BCP/ preCore regions)

DNA was extracted from serum samples according to the proteinase K protocol. Briefly, 200 μl of serum was added to 450 μl of mix containing 1 mg/ml proteinase K, 5mM Tris HCl (pH 8.5), 2.0% sodium dodecyl sulfate (SDS) and 25mM ethylenediaminetetraacetic acid (EDTA) and incubated at 37°C for 4 h. DNA was precipitated with 1 volume of absolute isopropanol in the presence of 20 μl of Dextran T500 and 1/10 volume of 3M NaAc (pH 4.7). DNA was recovered by centrifugation at 20,000 g for 15 min; pellets were washed with 70% ethanol, dried, and dissolved in 20 μl of water.

S gene was amplified with primers HBVS1 (sense, 5’ TCA CCA TAT TCT TGG GAA CAA GA 3’, 2821–2843) and HBVS2 (antisense, 5’ CAA AAG AAA ATT GGT AAC AGC GG 3’, 794–816). BCP and preCore regions were amplified by nested PCR using primers synthesized according to the consensus sequence of the pre-C region [32].

### HBV-DNA sequencing

PCR products covering the BCP/pC and S regions were purified by Qiagen columns (Qiagen, Germany), and direct sequencing was carried out using a 3730xl DNA Analyzer (Applied Biosystems, USA) in both amplification senses.

GenBank accession numbers for S region: DQ776245 to DQ776248; DQ776268 to DQ776272; EU366114 to EU366118; EU366123; EU366124; EU366129 to EU366133; EU366137; EU366138; FJ657518 to FJ657529; GU207481 to GU207485; GU207488 to GU207493; HM216215 to HM216257; HM216259 to HM216276; HM216278 to HM216286; KJ810909 to KJ810975; KJ843154 to KJ843218.

GenBank accession numbers for BCP and pC region: HM214716 to HM214756; HM216287 to HM216329; HM216331 to HM216348; HM216350 to HM216358; KJ810838 to KJ810908; KJ843154 to KJ843218.

### Amplification and sequencing of Basal Core Promoter and preCore gene

BCP and preCore regions were amplified by nested PCR using primers synthesized according to the consensus sequence of the pre-C region [32]. PCR products were purified by QIAgen columns (QIAgen), and direct sequencing was carried out in a 3730xl DNA Analyzer (Applied Biosystems) in both amplification senses (GenBank accession numbers: HM214716 to HM214756; HM216287 to HM216329; HM216331 to HM216348; HM216350 to HM216358; KJ810838 to KJ810908; KJ843154 to KJ843218).

### HBV Typing

Genotyping was assessed by phylogenetic analysis. Seventy one nucleotide sequences of S and BCP/preCore regions representing the different HBV genotypes were retrieved from GenBank and used as references. S and BCP/preCore sequences obtained in this study and HBV sequences from GenBank database were aligned with the ClustalX (v2.1) software [33] and edited with the BioEdit (v7.1.3.0) software [34].

Phylogenetic trees were constructed using the Maximum Likelihood method performed with the RAxML (v 8.0.24) program [35]. Evolutionary models were inferred according to the Akaike Information Criterion (AIC) statistics [36] obtained with the jModeltest (v2.1) software [37]. The robustness of the reconstructed phylogenies was evaluated by bootstrap analysis (1000 replicates).

In order to differentiate among subgenotypes, phylogenetic analyses were combined with the amino acid and nucleotide patterns characteristic of each subgenotype within the S, P and C open reading frames [38]; this was assessed by the VisSPA v1.6.2 program [39]. It was established that the amino acid pattern characteristic of each subgenotype would be formed by at least 90% of the amino acids present in the sequences from the group analyzed and in less than 10% of the samples in the reference group.

### Genetic Similarity

To determine the genetic similarity among HBV genotypes and subgenotypes, pairwise comparisons of 251 complete HBV genomes (53 genotype A, 115 genotype D, 55 subgenotype F1b and 28 subgenotype F4) retrieved from GenBank were analyzed with SimPlot software [40]. Distance plot and bootscanning analysis were performed using 200 nucleotide window size and 20 nucleotide increment steps.

### Statistical analysis

Fisher’s two-tailed exact test and the corrected X^2^ test were used to compare qualitative data. ANOVA and non-parametric tests (Mann-Whitney U and Kruskal-Wallis H) were used to compare quantitative variables. Results were expressed as mean ± SEM. Data analysis was performed by the statistical software package SPSS (version 10.0, SPSS, Inc., Chicago, USA). Significance was set as a p-value of less than 0.05.

Multivariate logistic regression analyses were used to determine the independent factors associated with the clinical course (acute/ chronic) and the HBeAg status of the chronic infections (HBeAg/ anti-HBe). Gender, age and genotype (A2, D, F1b, F4) were considered as variables. Dummy variables were created for the variable ‘genotype’ (with more than two classes). Analyses were performed with the Infostat vL software.

### Ethics Statement

This study was carried out according to the World Medical Association Declaration of Helsinki; it was approved by the Ethics Committee of the School of Pharmacy and Biochemistry, Buenos Aires University (Permit Number: 732575/2010) and written informed consent statements were signed by all patients.

## Results

In this cross-sectional study 234 HBsAg carriers were included: 66 had acute hepatitis and 168 were chronically infected, of whom 63 were HBeAg positive and 105 anti-HBe positive (Table 1).

**Table 1:** Age and gender distribution among different stages of infection.

	All cases	Acute(AHB)	Chronic(CHB)	
			HBeAg	anti-HBe
N	234	66	63	105
Age(mean±SD)	44.0±14.0	39.8±13.9*	43.3±16.7	47.3±11.5*
Male:Female(male to female ratio)	161:73(2.21)	53:13**(4.07)	45:18(2.50)	63:42**(1.50)

*AHB vs CHB anti-HBe positive p<0.001

**AHB vs CHB anti-HBe positive p<0.05.

The mean age of this cohort was 44.0 ± 14.0 years, being significantly younger than those patients with acute infection compared to those with CHB anti-HBe positive infections (p<0.001). Regarding gender, 73 patients (31.2%) were women and 161 were men (68.8%) (Table 1). The male to female ratio showed a significant difference between AHB (4.07) and CHB anti-HBe positive stages (1.50) (p<0.05).

### Genotype and subgenotype distribution

**Fig 1:**
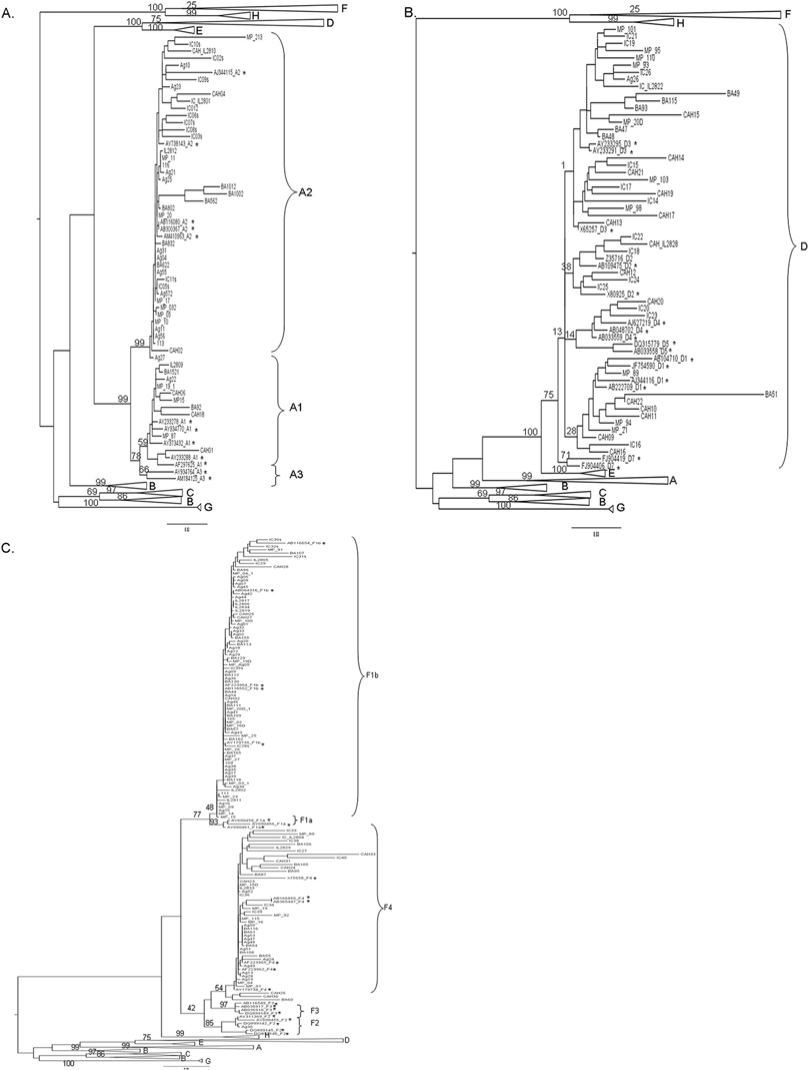
Phylogenetic-tree showing detailed genotype A (A), D (B) and F (C) branches. Maximum Likelihood phylogenetic-tree including the 234 Argentine HBV samples in this study, showing: (A) the whole tree with detailed genotype A branch; (B) genotype D branch; and (C) genotype F branch. Reference samples are marked with an asterisk. Genotypes and subgenotypes are indicated in capital letters. Numbers at each node correspond to bootstrap values obtained with 1000 replicates; only nodes corresponding to main groups are shown, for clarity purposes. Horizontal branches are drawn to scale.

HBV genotype (g) was determined by phylogenetic analyses (Fig 1). The overall genotype distribution was as follows: 127 F (54.3%), 52 A (22.2%), 49 D (20.9%) and other genotypes (4 B (1.7%), 1 C (0.4%), and 1 H (0.4%)) in the remaining patients.

Moreover, subgenotypes (sg) were identified within genotypes A (Fig 1A) and F (Fig 1C), whereas the phylogenetic signal of the BCP/pC and S regions was not enough to subgenotype genotype D samples (Fig 1B); consequently, these samples were analyzed as a whole.

Phylogenetic analysis was capable of differentiating among subgenotypes A1 (19.2%) and A2 (80.8%) from gA samples (Fig 1A). Therefore, further analyses were carried out with sgA2 samples.

Subgenotypying of gF samples was based on both phylogenetic analysis and nucleotide and amino acid comparisons along both S and P genes. The following nucleotide (nt) and amino acid (aa) patterns were characteristic of subgenotype F1b: nt T562, nt C1026, nt T1032 and aa rtL151 (P ORF); whereas those from subgenotype F4 were: nt A482, nt T493, aa L110 (S ORF), aa rtT118, aa rtH122 and aa rtN123 (P ORF).

These analyses showed that genotype F isolates could be subdivided into different subgenotypes; 80 belonged to sgF1b, 46 to sgF4 and 1 sample to sgF2a.

An uneven genotype distribution among AHB, CHB HBeAg and anti-HBe positive infections was observed (Table 2).

**Table 2:** HBV genotype distribution according to infection stage.

Genotype	All cases	A		D	F		Other*
Subgenotype		1	2		1b	4	
**Acute(%)**	66	2(3.0)	12(18.2)^a^	1(1.5)^a^	37(56.1)^a^	12(18.2)^a^	2(3.0)
**Chronic**							
Age(mean±SD)	45.8±14.5	46.9±12.7	46.5±12.3	46.1±12.8	46.2±14.0	42.5±15.5	46.6±17.5
HBeAg(%)	63	2(3.2)	15(23.8)	6(9.5)^b^	27(42.9)^b^	12(19.0)	1(1.6)
anti-HBe(%)	105	6(5.7)	15(14.3)^c^	42(40.0)^c^	16(15.2)^c^	22(21.0)^c^	4(3.8)
HBeAg positivity % in CHB	37.5	25.0	50.0^d^	12.5^d^	62.8^d^	35.3^d^	20.0

*Other genotypes: B, C, F2a and H.

^a^ F1b vs. A, D and F4 p<0.05, A vs. D p<0.05

^b^ F1b vs. D p<0.05

^c^ D vs. A, F1b and F4 p<0.001, F1b vs. A and F4 p<0.05

^d^ F1b vs. D and F4 p<0.05; A vs. D p<0.05.

In AHB infection, subgenotype F1b was the most prevalent (56.1%), followed by sgA2 (18.2%), sgF4 (18.2%) and D (1.5%) (Table 2). This distribution was similar, but less pronounced, among CHB HBeAg positive patients, where subgenotype F1b was the most frequent genotype (42.9%) and genotype D was the least frequent one (9.5%). The opposite distribution pattern was observed in CHB anti-HBe positive patients, where gD was the most prevalent (40.0%), whereas sgF1b was the least prevalent (15.2%).

Moreover, in chronic infections, a significantly different HBeAg positivity rate among genotypes was observed, being higher in sgF1b (62.8%), intermediate in sgA2 (50.0%) and sgF4 (35.3%), and lower in gD (12.5%).

No significant differences were observed among the ages of patients infected with different HBV genotypes (Table 2).

### Multivariate analysis

Using a multivariate analysis with age, gender and genotype as variables, only age and genotype were independently associated with the acute/chronic course of infection. As in univariate analysis, advanced ages were associated with chronic infections (Odd Ratio = 1.03, p = 0.018). Specifically, sgA2, sgF4 and gD were more associated with the chronic course of infection than sgF1b (sgA2: OR = 2.17, p = 0.049; sgF4: OR = 2.52, p = 0.027; gD: OR = 35.13, p<0.001).

In addition, age and genotype were independently associated with the HBeAg/ anti-HBe stage of chronic infections. Advanced age was associated with CHB anti-HBe positive stage (OR = 1.03, p = 0.026), whereas sgF1b was more associated with CHB HBeAg positive stage (sgF4: OR = 3.52, p = 0.004; gD: OR = 8.87, p<0.001).

### Basal Core Promoter and preCore mutations distribution

Mutations modulating HBeAg expression were observed in all HBV infection stages, being more prevalent in CHB anti-HBe positive patients (92.4%) than in AHB (24.2%) and CHB HBeAg positive patients (20.6%) (Table 3).

**Table 3:** Mutation profiles of HBV BCP/pC regions in different viral genotypes and infection stages.

Genotype	All cases	A1	A2	D	F1b	F4	Other
**Acute**	**66**	**2**	**12**	**1**	**37**	**12**	**2**
A1762T/G1764A	14(21.2)	0(0.0)	3(25.0)	0(0.0)	11(29.7)	0(0.0)	0(0.0)
G1896A	2(3.0)	0(0.0)	0(0.0)	1(100.0)	1(2.7)	0(0.0)	0(0.0)
Other*	1(1.5)	0(0.0)	0(0.0)	0(0.0)	0(0.0)	1(8.3)	0(0.0)
Wild type strains	50(75.8)	2(100.0)	9(75.0)	0(0.0)	26(70.3)	11(91.7)	2(100.0)
**Chronic**							
**HBeAg**	**63**	**2**	**15**	**6**	**27**	**12**	**1**
A1762T/G1764A(%)	11(17.5)	0(0.0)	1(6.7)	2(33.3)	7(25.9)	0(0.0)	1(100.0)
G1896A(%)	2(3.2)	0(0.0)	0(0.0)	1(16.7)	0(0.0)	1(8.3)	0(0.0)
Other*(%)	0(0.0)	0(0.0)	0(0.0)	0(0.0)	0(0.0)	0(0.0)	0(0.0)
Wild type strains	50(79.3)	2(100.0)	14(93.3)	3(50.0)	20(74.1)	11(91.7)	0(0.0)
**anti-HBe**	**105**	**6**	**15**	**42**	**16**	**22**	**4**
A1762T/G1764A(%)	41(39.0)	2(33.3)	6(40.0)^a^	14(33.3)^a^	12(75.0)^a^	7(31.8)^a^	0(0.0)
G1896A(%)	58(55.2)	1(16.7)	0(0.0)^b^	34(81.0)^b^	5(31.3)^b^	16(72.7)^b^	2(50.0)
Other*(%)	35(33.3)	4(66.7)	10(66.7)^c^	10(23.8)^c^	6(37.5)	4(18.2)^c^	1(25.0)
Wild type strains	8(7.6)	0(0.0)	2 (13.3)	0(0.0)	3(18.8)	2(9.1)	1(25.0)

*Other mutations in the preCore region that abolish HBeAg expression. No significant difference was observed in the frequency of mutations, in AHB and CHB HBeAg positive patients, among genotypes.

^a^ F1b vs. A2, D and F4 p<0.05

^b^ D vs. A2 and F1b p<0.001, F4 vs A2 and F1b p<0.05

^c^ A2 vs. D and F4 p<0.05.

Among AHB and CHB HBeAg positive infections, mutations were more frequently found in the BCP region (21.2 and 17.5%) than in the pC region (4.5 and 3.2%).

In anti-HBe positive chronic infections, mutations affecting HBeAg expression were observed in 97 out of 105 (92.4%) samples, and more than one mutation was found in 30% of them. In the preCore region, G1896A was the most common mutation (55.2%), whereas other mutations that prevent HBeAg synthesis, such as those affecting the preCore initiation codon (nt 1814–1816), mutations (C1817T, G1897A), insertions and deletions that create a premature stop codon, were observed in a lower frequency (33.3%).

### BCP and pC mutations by genotype and infection stage

In spite of the low prevalence of mutations in AHB infections (25.7%), those subgenotypes more frequently observed in this stage, sgA2 and sgF1b, had the double mutation A1762T/G1764A, while gD and sgF4 did not mutate these positions (Table 3). In CHB HBeAg positive stage, there was no significant difference in the frequency of BCP or pC mutations among different genotypes.

In anti-HBe positive patients, mutations were biased by genotype. The double mutation A1762T/G1764A was more frequently found in sgF1b infections (75.0%) than in sgA2, gD and sgF4 (40.0, 33.3 and 31.8%, respectively). In the preCore region, gD and sgF4 had higher frequencies of G1896A mutation (81.0 and 72.7%, respectively) compared to sgA2 and sgF1b (0.0 and 31.3%). Interestingly, in sgA2, other mutations that abrogate HBeAg expression in the preCore region were the most prevalent (66.7%).

In brief, those patients infected with sgF4 and gD mutated G1896A more frequently than A1762T/G1764A (p = 0.007 and p<0.001 respectively), whereas those patients carrying sgF1b and sgA2 had the opposite mutation pattern, showing higher rates of mutations in positions 1762 and 1764 than in 1896 (p = 0.013 and p = 0.010 respectively).

### Nucleotide similarity among HBV genotypes

It is widely accepted that the G1896A mutation rate is closely related to the viral genotype. This mutation is rarely selected in genotypes carrying 1858C (gA, F2, F3 and H), while it has been frequently observed in those genotypes carrying 1858T (B, D, E and G).

This paradigm is based on structural principles. The HBV encapsidation signal, essential for pregenomic RNA encapsidation and viral replication, overlaps almost the entire precore region. In the RNA, the signal forms a double stem-loop structure and nucleotide 1896 is base-paired with nucleotide 1858. The G1896A mutation rate observed in sgF1b (carrying 1858T) was unexpectedly low, displaying a mutation pattern more similar to genotype A (1858C) than genotypes D and F4 (1858T).

This result prompted us to perform further analysis in order to identify viral polymorphisms, other than position 1858, that may be involved in the molecular mechanisms of HBeAg seroconversion.

In order to map nucleotide similarities among the different genotypes along the whole viral genome a SimPlot analysis was performed using a data set of 251 full length genome sequences retrieved from GenBank, representing strains from genotype A(n = 53), D (n = 115), F1b (n = 55) and F4 (n = 28).

**Fig 2:**
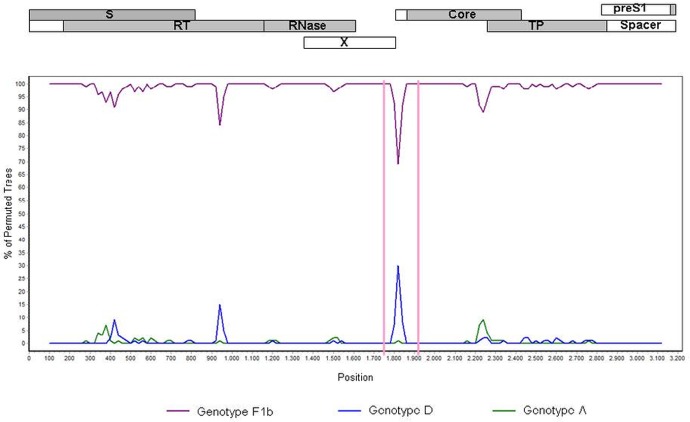
Bootscan plot. Nucleotide similarity comparison between genotype F4 against genotypes F1b (violet line), D (blue line) and A (green line). The graph was generated using Simplot ver. 3.5.1 with window size 200 bp, step size 20 bp, gap-strip off, 100 bootstrap replicates, Kimura transition/transversion ratio:2 and neighbor-joining. The genomic regions are shaded in gray on the top of the figure, which shows the genomic arrangement of the HBV open reading frames. Numbers on the x axis denote nucleotide positions from the start of EcoR1 restriction site.

Along the whole genome, subgenotype F1b showed, as expected, the highest degree of similarity when compared with subgenotype F4. Nevertheless, in the region encompassing the nucleotides 1820 ± 100, a higher degree of similarity and an increase in phylogenetic association between F4 and D (Fig 2), as well as between F1b and A (data not shown), was observed.

**Fig 3:**
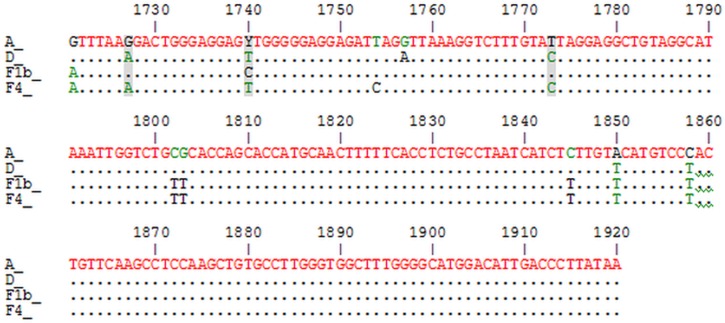
Alignment of consensus sequences spanning 1820 ± 100 nucleotide region of different genotypes. Non-identical nucleotides for genotypes A, D, F1b and F4 are shown. Nucleotides shared between genotype A and F1b are shaded.

The alignment of the consensus sequences spanning the 1820 ± 100 nucleotide region showed a high degree of conservation in this region among genotypes. Nonetheless, in the BCP region there are three identical nucleotide positions in genotypes A and F1b (1727G, 1740C and 1773T), different from those present in genotypes D and F4 (1727A, 1740T, 1773C). These polymorphisms are spotted in the reading frame of the X protein (Fig 3).

In order to assess the role of 1727, 1740 and 1773 polymorphisms in the mutation pattern of 1896 position, the frequency of G1896A mutation was determined in those samples carrying 1858T. Samples with 1727A, 1740T and 1773C (D and F4) were more prone to select G1896A mutation than those with 1727G, 1740C and 1773T (F1b) (p<0.05) (Table 4).

**Table 4:** Frequency of G1896A mutation according to the polymorphisms at nucleotides 1727, 1740 and 1773.

		Position 1896		p value
Position	Polymorphism	G	A	
1727	G(%)	12(54.5)	10(45.5)	0.013
	A(%)	13(22.4)	45(77.6)	
1740	C(%)	11(68.7)	5(31.3)	<0.001
	T(%)	14(21.8)	50(78.2)	
1773	T(%)	13(56.5)	10(43.5)	0.003
	C(%)	12(21.0)	45(79.0)	

## Discussion

There is growing evidence that HBV genotypes may play a role in causing different disease profiles in chronic hepatitis B infection [41]. However, most of the information on the clinical significance of HBV genotypes has been based on studies performed in Asia or in Europe, with patients infected with genotypes B and C or A and D, respectively. Added to the fact that comparisons have been made between two genotypes, there is a paucity of data on the clinical course of patients with other genotypes, different from A-D [38,42,43].

The present study highlights the differences in genotype and subgenotype distribution among subjects with acute, chronic HBeAg positive and chronic anti-HBe positive infections.

The difference in genotype distribution between HBeAg positive and anti-HBe positive chronic infections suggests a different seroconversion rate among genotypes and subgenotypes (D>>F4>A2>F1b). This might be relevant since different studies have observed that delayed HBeAg seroconversion is associated with a more severe clinical course of infection [41,44–46]. This highlights the implication of the viral genotype in the progression of the infection.

In line with our findings, the few studies that have assessed subgenotype F1b suggest that this subgenotype has a worse clinical outcome than other genotypes [42,47]. This genotype’s behavior could be compared to that of genotype C, observed in different studies performed in Asia, where it showed a higher rate of HBeAg positivity and a worse clinical outcome of the chronic infection compared to those infections caused by genotype B [31,48].

On the other hand, the similar genotype distribution in acute and HBeAg positive chronic infections could be explained by the fact that transmission of HBV most probably occurs during the HBeAg positive stage [49,50]. It has been previously reported that in the latter stage, viral loads are usually higher than in the anti-HBe positive stage of infection [51–54].

Regarding mutations in the BCP and pC regions, these were more prevalent in the anti-HBe positive stage than in the other two stages of infection. However, in those AHB and CHB HBeAg positive patients infected with viral variants carrying mutations in these regions, BCP mutations were more frequently found (19.4%) than pC mutations (3.9%). This is consistent with the fact that BCP mutations down-regulate HBeAg expression, while pC mutations abolish HBeAg synthesis [55].

Furthermore, in the anti-HBe positive stage, a bias of mutations among genotypes was observed. Mutations in the BCP were more frequently found in subgenotype F1b (75.0%) than in A2, D and F4 (40.0, 33.3 and 31.8%, respectively). *In vitro* studies have shown that mutation A1762T/G1764A does not abrogate HBeAg expression but decreases its levels, while concomitantly increasing viral replication [15,56]. Thus, the lower seroconversion rate observed in genotype F might be explained by the higher frequency of BCP mutations in this genotype.

Overall, these findings suggest that intrinsic biological features of each genotype may lead to a longer HBeAg positive stage and therefore to different implications in the progression of the infection.

The difference in the mutation pattern among genotypes was initially described in the 90s after the identification of genotypes A to D, when it was observed that the occurrence of G1896A was restricted to HBV genotypes with T at nucleotide 1858 [57–59]. Given the fact that pC region overlaps the encapsidation signal, which is essential for efficient replication, those genotypes with T1858 would tend to mutate G1896A in order to increase stability of the stem loop in the encapsidation signal structure.

Since subgenotypes F1b and F4 carry T1858, it would be expected that G1896A mutation would predominate in both subgenotypes; however, this was only observed in subgenotype F4. The bias in the mutation pattern between these two subgenotypes has been previously overlooked, probably because few studies have discriminated between these subgenotypes.

This controversy has also been observed in genotype C; although most subgenotypes carry 1858T, there is a strong bias toward using BCP mutations. On the contrary, subtypes B2 and B3 tend to acquire codon 28 mutations, even though their core promoters and preCore/core genes are derived from genotype C [31]. Furthermore, despite the fact that all subgenotypes D have T1858, only subgenotypes D1 and D7 have a tendency to mutate G1896A [60].

The differences in the molecular features of distinct subgenotypes from genotypes B, C, D and F [61–66] highlight the relevance of differentiating HBV subgenotypes when analyzing their implications in the progression of the infection.

In summary, those genotypes carrying 1858C seem to prevent G1896A mutation; however, T1858 polymorphism seems to be necessary but not sufficient to promote G1896A substitution.

These observations suggest that the choice of the mechanism that a given genotype uses to regulate HBeAg expression is not fully explained by the encapsidation signal structure. Furthermore, several *in vitro* studies have shown that there is no strict relationship between the stability of this signal and the replication capacity [14,67–69]. Overall, these findings indicate that sequences outside the encapsidation signal may influence the mechanism of choice.

On the other hand, the high prevalence of mutations other than G1896A in the preCore region in subgenotype A2 indicates that during virus-host interaction, the virus explores different molecular alternatives to regulate HBeAg expression.

The nucleotide alignment of the region encompassing nucleotides 1720 to 1920 suggests that the similarity observed between genotype A and F1b, and between F4 and D, seems to lay on three nucleotide positions (1729, 1740 and 1773). The fact that these nucleotide polymorphisms are synonymous in the open reading frame of X protein implies that the differences observed among genotypes might not be due to changes at protein X but at a regulatory or RNA conformational level.

Nucleotide 1773 is in the phi region, a cis-acting element which has been proposed to be involved in minus-strand DNA synthesis, as it may mediate the translocation of the viral polymerase during replication [70]. Nucleotides in the phi region base pair with nucleotides in the 5’ half of the encapsidation signal; for instance, position 1773 pairs with 1876 [71]. The implication of these positions and the mechanism by which they could be related to mutations regulating HBeAg expression, and thereby to seroconversion, should be cause for further elucidations.

In conclusion, our results show a different HBeAg positivity rate among genotypes/subgenotypes (F1b>A>F4>>D), which could imply a difference in the duration of the HBe positive stage, with its consequent implication in the progression of liver disease. This finding supports the uneven distribution of genotypes between primary and chronic infections and its ensuing epidemiological implications.

Finally, we identified three nucleotide positions outside the encapsidation signal that could contribute to the underlying mechanism related to HBeAg seroconversion in those HBV subgenotypes that displaying 1858T prevented the mutation in the 1896 position.
